# Supplementary material for: Discovery and application of insertion-deletion (INDEL) polymorphisms for QTL mapping of early life-history traits in Atlantic salmon
Source: BMC Genomics. 2010 Mar 8;11:156. doi: 10.1186/1471-2164-11-156 (PMC2838853; doi:10.1186/1471-2164-11-156)
Supplement: Additional file 2 — Information on developed 76 locus single-run INDEL panel in Atlantic salmon. Information on fluorescence labeling, primer concentrations, PCR pooling and links to alignments, INDEL motifs and GENESCAN (Burge and Karlin 1997) predictions of genes/exons are available in html format. [file 1471-2164-11-156-S2.ZIP › Additionalfile2/snpsummary2456.html]

```
Cluster 450 Contig 1

prev  Summary    Contig List  next
```

Size of Consensus sequence = 948

Number of sequences = 69

Minimum redundancy = 6

Key

A gi|117857711|gb|EG930407.1|EG930407 EST\_ssal\_evf\_32524 ssalevf mixed\_tissue Salmo salar cDNA Salmo salar cDNA clone ssal\_evf\_543\_085\_fwd 3', mRNA sequence  
B gi|117848108|gb|EG920804.1|EG920804 EST\_ssal\_evf\_80 ssalevf mixed\_tissue Salmo salar cDNA Salmo salar cDNA clone ssal\_evf\_001\_043\_fwd 3', mRNA sequence  
C gi|45314089|gb|CK884458.1|CK884458 SGP148343 Atlantic salmon Intestine cDNA library Salmo salar cDNA clone T6-0795 5', mRNA sequence  
D gi|29314983|gb|CB503757.1|CB503757 ssalplnb512053\_rev gut Salmo salar cDNA, mRNA sequence  
E gi|84982102|gb|DW532452.1|DW532452 EST\_ssal\_plnb\_1088 plnb Salmo salar cDNA clone ssal\_plnb\_009\_051\_rev 5', mRNA sequence  
F gi|84983791|gb|DW534141.1|DW534141 EST\_ssal\_plnb\_2777 plnb Salmo salar cDNA clone ssal\_plnb\_020\_037\_rev 5', mRNA sequence  
G gi|89828849|gb|DY691784.1|DY691784 EST\_ssal\_plnb\_4694 ssalplnb mixed\_tissue Salmo salar cDNA Salmo salar cDNA clone ssal\_plnb\_024\_345\_rev 5', mRNA sequence  
H gi|15845034|gb|BG935206.1|BG935206 SL1-0517 Atlantic Salmon liver Salmo salar cDNA clone SL1-0517 similar to Calcium and integrin binding protein CIB, mRNA sequence  
I gi|84982929|gb|DW533279.1|DW533279 EST\_ssal\_plnb\_1915 plnb Salmo salar cDNA clone ssal\_plnb\_015\_012\_rev 5', mRNA sequence  
J gi|117456077|gb|EG788296.1|EG788296 EST\_ssal\_evd\_8786 ssalevd thymus Salmo salar cDNA Salmo salar cDNA clone ssal\_evd\_510\_188\_fwd 3', mRNA sequence  
K gi|117465172|gb|EG797391.1|EG797391 EST\_ssal\_evd\_52400 ssalevd thymus Salmo salar cDNA Salmo salar cDNA clone ssal\_evd\_570\_242\_fwd 3', mRNA sequence  
L gi|117566990|gb|EG887966.1|EG887966 EST\_ssal\_evf\_46070 ssalevf mixed\_tissue Salmo salar cDNA Salmo salar cDNA clone ssal\_evf\_561\_219\_fwd 3', mRNA sequence  
M gi|84992177|gb|DW542527.1|DW542527 EST\_ssal\_rgb2\_6946 rgb2 Salmo salar cDNA clone ssal\_rgb2\_512\_152\_rev 5', mRNA sequence  
N gi|117819628|gb|EG892324.1|EG892324 EST\_ssal\_evf\_49993 ssalevf mixed\_tissue Salmo salar cDNA Salmo salar cDNA clone ssal\_evf\_566\_334\_fwd 3', mRNA sequence  
O gi|117521728|gb|EG853455.1|EG853455 EST\_ssal\_eve\_14302 ssaleve thyroid Salmo salar cDNA Salmo salar cDNA clone ssal\_eve\_519\_164\_fwd 3', mRNA sequence  
P gi|117516718|gb|EG848477.1|EG848477 EST\_ssal\_eve\_6021 ssaleve thyroid Salmo salar cDNA Salmo salar cDNA clone ssal\_eve\_507\_070\_fwd 3', mRNA sequence  
Q gi|89878996|gb|DY735119.1|DY735119 EST\_ssal\_rgb2\_90858 ssalrgb2 mixed\_tissue Salmo salar cDNA Salmo salar cDNA clone ssal\_rgb2\_648\_292\_rev 5', mRNA sequence  
R gi|117517400|gb|EG849159.1|EG849159 EST\_ssal\_eve\_6635 ssaleve thyroid Salmo salar cDNA Salmo salar cDNA clone ssal\_eve\_508\_003\_fwd 3', mRNA sequence  
S gi|89828754|gb|DY691733.1|DY691733 EST\_ssal\_plnb\_4643 ssalplnb mixed\_tissue Salmo salar cDNA Salmo salar cDNA clone ssal\_plnb\_024\_313\_rev 5', mRNA sequence  
T gi|117524329|gb|EG856056.1|EG856056 EST\_ssal\_eve\_16643 ssaleve thyroid Salmo salar cDNA Salmo salar cDNA clone ssal\_eve\_522\_229\_fwd 3', mRNA sequence  
U gi|57124461|gb|CX355902.1|CX355902 ssalrgb536360\_rev\_0 mixed\_tissue Salmo salar cDNA, mRNA sequence  
V gi|117460015|gb|EG792234.1|EG792234 EST\_ssal\_evd\_12330 ssalevd thymus Salmo salar cDNA Salmo salar cDNA clone ssal\_evd\_515\_136\_fwd 3', mRNA sequence  
W gi|117502292|gb|EG834088.1|EG834088 EST\_ssal\_eve\_45972 ssaleve thyroid Salmo salar cDNA Salmo salar cDNA clone ssal\_eve\_562\_205\_fwd 3', mRNA sequence  
X gi|117828499|gb|EG901195.1|EG901195 EST\_ssal\_evf\_2809 ssalevf mixed\_tissue Salmo salar cDNA Salmo salar cDNA clone ssal\_evf\_502\_016\_fwd 3', mRNA sequence  
Y gi|117563891|gb|EG884867.1|EG884867 EST\_ssal\_evf\_43281 ssalevf mixed\_tissue Salmo salar cDNA Salmo salar cDNA clone ssal\_evf\_557\_322\_fwd 3', mRNA sequence  
Z gi|117866591|gb|EG939287.1|EG939287 EST\_ssal\_evf\_22926 ssalevf mixed\_tissue Salmo salar cDNA Salmo salar cDNA clone ssal\_evf\_530\_080\_fwd 3', mRNA sequence  
a gi|117837272|gb|EG909968.1|EG909968 EST\_ssal\_evf\_12128 ssalevf mixed\_tissue Salmo salar cDNA Salmo salar cDNA clone ssal\_evf\_514\_266\_fwd 3', mRNA sequence  
b gi|117427144|gb|EG759368.1|EG759368 EST\_ssal\_sjb\_4201 ssalsjb mixed\_tissue Salmo salar cDNA Salmo salar cDNA clone ssal\_sjb\_011\_056\_fwd 3', mRNA sequence  
c gi|117489555|gb|EG821772.1|EG821772 EST\_ssal\_evd\_23576 ssalevd thymus Salmo salar cDNA Salmo salar cDNA clone ssal\_evd\_530\_226\_fwd 3', mRNA sequence  
d gi|117537604|gb|EG869049.1|EG869049 EST\_ssal\_eve\_30336 ssaleve thyroid Salmo salar cDNA Salmo salar cDNA clone ssal\_eve\_541\_076\_fwd 3', mRNA sequence  
e gi|117819924|gb|EG892620.1|EG892620 EST\_ssal\_evf\_50259 ssalevf mixed\_tissue Salmo salar cDNA Salmo salar cDNA clone ssal\_evf\_567\_087\_rev 5', mRNA sequence  
f gi|117819319|gb|EG892015.1|EG892015 EST\_ssal\_evf\_49715 ssalevf mixed\_tissue Salmo salar cDNA Salmo salar cDNA clone ssal\_evf\_566\_192\_fwd 3', mRNA sequence  
g gi|117819320|gb|EG892016.1|EG892016 EST\_ssal\_evf\_49716 ssalevf mixed\_tissue Salmo salar cDNA Salmo salar cDNA clone ssal\_evf\_566\_192\_rev 5', mRNA sequence  
h gi|117825266|gb|EG897962.1|EG897962 EST\_ssal\_evf\_2518 ssalevf mixed\_tissue Salmo salar cDNA Salmo salar cDNA clone ssal\_evf\_501\_247\_rev 5', mRNA sequence  
i gi|117825277|gb|EG897973.1|EG897973 EST\_ssal\_evf\_2519 ssalevf mixed\_tissue Salmo salar cDNA Salmo salar cDNA clone ssal\_evf\_501\_247\_fwd 3', mRNA sequence  
j gi|117529462|gb|EG861103.1|EG861103 EST\_ssal\_eve\_54284 ssaleve thyroid Salmo salar cDNA Salmo salar cDNA clone ssal\_eve\_573\_366\_fwd 3', mRNA sequence  
k gi|117529463|gb|EG861104.1|EG861104 EST\_ssal\_eve\_54285 ssaleve thyroid Salmo salar cDNA Salmo salar cDNA clone ssal\_eve\_573\_366\_rev 5', mRNA sequence  
l gi|84982736|gb|DW533086.1|DW533086 EST\_ssal\_plnb\_1722 plnb Salmo salar cDNA clone ssal\_plnb\_013\_081\_rev 5', mRNA sequence  
m gi|84982737|gb|DW533087.1|DW533087 EST\_ssal\_plnb\_1723 plnb Salmo salar cDNA clone ssal\_plnb\_013\_081\_fwd 3', mRNA sequence  
n gi|117848109|gb|EG920805.1|EG920805 EST\_ssal\_evf\_81 ssalevf mixed\_tissue Salmo salar cDNA Salmo salar cDNA clone ssal\_evf\_001\_043\_rev 5', mRNA sequence  
o gi|84982930|gb|DW533280.1|DW533280 EST\_ssal\_plnb\_1916 plnb Salmo salar cDNA clone ssal\_plnb\_015\_012\_fwd 3', mRNA sequence  
p gi|29317248|gb|CB506022.1|CB506022 ssalmgd504227 gut Salmo salar cDNA, mRNA sequence  
q gi|117516719|gb|EG848478.1|EG848478 EST\_ssal\_eve\_6022 ssaleve thyroid Salmo salar cDNA Salmo salar cDNA clone ssal\_eve\_507\_070\_rev 5', mRNA sequence  
r gi|89828756|gb|DY691734.1|DY691734 EST\_ssal\_plnb\_4644 ssalplnb mixed\_tissue Salmo salar cDNA Salmo salar cDNA clone ssal\_plnb\_024\_313\_fwd 3', mRNA sequence  
s gi|89828851|gb|DY691785.1|DY691785 EST\_ssal\_plnb\_4695 ssalplnb mixed\_tissue Salmo salar cDNA Salmo salar cDNA clone ssal\_plnb\_024\_345\_fwd 3', mRNA sequence  
t gi|117857710|gb|EG930406.1|EG930406 EST\_ssal\_evf\_32523 ssalevf mixed\_tissue Salmo salar cDNA Salmo salar cDNA clone ssal\_evf\_543\_085\_rev 5', mRNA sequence  
u gi|84982103|gb|DW532453.1|DW532453 EST\_ssal\_plnb\_1089 plnb Salmo salar cDNA clone ssal\_plnb\_009\_051\_fwd 3', mRNA sequence  
v gi|117456076|gb|EG788295.1|EG788295 EST\_ssal\_evd\_8785 ssalevd thymus Salmo salar cDNA Salmo salar cDNA clone ssal\_evd\_510\_188\_rev 5', mRNA sequence  
w gi|117524328|gb|EG856055.1|EG856055 EST\_ssal\_eve\_16642 ssaleve thyroid Salmo salar cDNA Salmo salar cDNA clone ssal\_eve\_522\_229\_rev 5', mRNA sequence  
x gi|117866592|gb|EG939288.1|EG939288 EST\_ssal\_evf\_22927 ssalevf mixed\_tissue Salmo salar cDNA Salmo salar cDNA clone ssal\_evf\_530\_080\_rev 5', mRNA sequence  
y gi|24342636|gb|CA041716.1|CA041716 ssalplnb512053 gut Salmo salar cDNA, mRNA sequence  
z gi|84983792|gb|DW534142.1|DW534142 EST\_ssal\_plnb\_2778 plnb Salmo salar cDNA clone ssal\_plnb\_020\_037\_fwd 3', mRNA sequence  
A gi|117489556|gb|EG821773.1|EG821773 EST\_ssal\_evd\_23577 ssalevd thymus Salmo salar cDNA Salmo salar cDNA clone ssal\_evd\_530\_226\_rev 5', mRNA sequence  
B gi|24387298|gb|CA057055.1|CA057055 ssalrgb536360 mixed\_tissue Salmo salar cDNA, mRNA sequence  
C gi|89878997|gb|DY735120.1|DY735120 EST\_ssal\_rgb2\_90859 ssalrgb2 mixed\_tissue Salmo salar cDNA Salmo salar cDNA clone ssal\_rgb2\_648\_292\_fwd 3', mRNA sequence  
D gi|117521729|gb|EG853456.1|EG853456 EST\_ssal\_eve\_14303 ssaleve thyroid Salmo salar cDNA Salmo salar cDNA clone ssal\_eve\_519\_164\_rev 5', mRNA sequence  
E gi|117837273|gb|EG909969.1|EG909969 EST\_ssal\_evf\_12129 ssalevf mixed\_tissue Salmo salar cDNA Salmo salar cDNA clone ssal\_evf\_514\_266\_rev 5', mRNA sequence  
F gi|117517399|gb|EG849158.1|EG849158 EST\_ssal\_eve\_6634 ssaleve thyroid Salmo salar cDNA Salmo salar cDNA clone ssal\_eve\_508\_003\_rev 5', mRNA sequence  
G gi|117819627|gb|EG892323.1|EG892323 EST\_ssal\_evf\_49992 ssalevf mixed\_tissue Salmo salar cDNA Salmo salar cDNA clone ssal\_evf\_566\_334\_rev 5', mRNA sequence  
H gi|24339509|gb|CA039141.1|CA039141 ssalnwh014051 whole Salmo salar cDNA, mRNA sequence  
I gi|117566991|gb|EG887967.1|EG887967 EST\_ssal\_evf\_46071 ssalevf mixed\_tissue Salmo salar cDNA Salmo salar cDNA clone ssal\_evf\_561\_219\_rev 5', mRNA sequence  
J gi|117563892|gb|EG884868.1|EG884868 EST\_ssal\_evf\_43282 ssalevf mixed\_tissue Salmo salar cDNA Salmo salar cDNA clone ssal\_evf\_557\_322\_rev 5', mRNA sequence  
K gi|117828488|gb|EG901184.1|EG901184 EST\_ssal\_evf\_2808 ssalevf mixed\_tissue Salmo salar cDNA Salmo salar cDNA clone ssal\_evf\_502\_016\_rev 5', mRNA sequence  
L gi|117460016|gb|EG792235.1|EG792235 EST\_ssal\_evd\_12331 ssalevd thymus Salmo salar cDNA Salmo salar cDNA clone ssal\_evd\_515\_136\_rev 5', mRNA sequence  
M gi|117423799|gb|EG756023.1|EG756023 EST\_ssal\_sjb\_4823 ssalsjb mixed\_tissue Salmo salar cDNA Salmo salar cDNA clone ssal\_sjb\_012\_026\_rev 5', mRNA sequence  
N gi|89852367|gb|DY708490.1|DY708490 EST\_ssal\_rgb2\_64229 ssalrgb2 mixed\_tissue Salmo salar cDNA Salmo salar cDNA clone ssal\_rgb2\_603\_258\_fwd 3', mRNA sequence  
O gi|89852368|gb|DY708491.1|DY708491 EST\_ssal\_rgb2\_64230 ssalrgb2 mixed\_tissue Salmo salar cDNA Salmo salar cDNA clone ssal\_rgb2\_603\_258\_rev 5', mRNA sequence  
P gi|117423800|gb|EG756024.1|EG756024 EST\_ssal\_sjb\_4824 ssalsjb mixed\_tissue Salmo salar cDNA Salmo salar cDNA clone ssal\_sjb\_012\_026\_fwd 3', mRNA sequence  
Q gi|117502293|gb|EG834089.1|EG834089 EST\_ssal\_eve\_45973 ssaleve thyroid Salmo salar cDNA Salmo salar cDNA clone ssal\_eve\_562\_205\_rev 5', mRNA sequence

5 SNPs detected

A B C D E F G H I J K L M N O P Q R S T U V W X Y Z a b c d e f g h i j k l m n o p q r s t u v w x y z A B C D E F G H I J K L M N O P Q  cosegregation weighted

125 - - - - . - - A - - - - A - - - A - . - . A - - - - - A A - . . . . . . . . . . . . . . . . . . . . . . . . . . . . . . . . . . . . A A .   3/5 25.22
126 - - - - . - - C - - - - C - - - C - . - . C - - - - - C C - . . . . . . . . . . . . . . . . . . . . . . . . . . . . . . . . . . . . C C .   3/5 25.22
127 - - - - . - - A - - - - A - - - A - . - . A - - - - - A A - . . . . . . . . . . . . . . . . . . . . . . . . . . . . . . . . . . . . A A .   3/5 25.22
295 T T T T T T T G T T T T T T T T T T T T T G T T T T T G T T . . . . . . . . . . T G T . . . . T T T T T T T T T T T T . T T T G G G G G T   1/5 15.65
537 . G G G G G G G G G G G A G G G A G G G A G G G G G G G A G . . . G G G G G G G G G G G G G G G G G G G A A A G G G G G G G G G G G G G G   1/5 18.84
